# Myocardial Perfusion Scintigraphy after Percutaneous Coronary Intervention in Asymptomatic Patients: Useful or Futile?

**DOI:** 10.5935/abc.20180237

**Published:** 2018-12

**Authors:** Gabriel Blacher Grossman

**Affiliations:** 1 Hospital Moinhos de Vento, Porto Alegre, RS - Brazil; 2 Cardionuclear - Instituto de Cardiologia, Porto Alegre, RS - Brazil

**Keywords:** Coronary Artery Disease/radionuclide imaging, Myocardial Revascularization, Percutaneous Coronary Intervention, Myocardial Ischemia

Myocardial perfusion scintigraphy (MPS) is a well-established non-invasive method for the evaluation of patients with suspected ischemic heart disease or with coronary artery disease (CAD).^1^ Its major diagnostic indication is in the assessment of patients with intermediate likelihood of CAD,^2^ with the diagnostic value being difficult to be dissociated from the prognostic information obtained with the method. Through several criteria validated in the literature, such as the extent of ischemia, the patient's risk of presenting cardiovascular events in the future^3^ can be assessed. In patients with established CAD, MPS has an important role in the evaluation of symptoms suggestive of myocardial ischemia, and can also assess the risk of non-fatal myocardial infarction and cardiac death. Although the value of quantification of ischemia has been the subject of debate in recent years, it is undeniable that in clinical practice it can assist in therapeutic decision-making.^4,5^

MPS may be useful in the evaluation of patients undergoing surgical or percutaneous revascularization procedures, especially if the patient has symptoms. Although MPS can be indicated in asymptomatic patients after 2 years of percutaneous coronary intervention (PCI) or 5 years of surgical procedure,^6^ few studies in the literature have analyzed the adequate time to perform the functional study in asymptomatic patients, and the clinical impact of this information. Cardiologic practice often contradicts what is recommended, and it is not uncommon to evaluate asymptomatic patients in a shorter period than that suggested in the literature.

In this edition of the Arquivos Brasileiros de Cardiologia, de Andrade et al.^7^ evaluated the prognostic value and clinical use of MPS in asymptomatic patients after PCI.^7^ The authors conducted a retrospective study evaluating 647 patients that were submitted to MPS after PCI. Fifty three percent of the patients presented abnormal MPS (30% abnormal with ischemia and 23% abnormal without ischemia). The annual rate of death was higher in those with abnormal perfusion without ischemia compared to the groups with ischemia and with normal MPS (3.3% x 2% x 1.2%, p = 0,021). The annual revascularization rate was 10.3% in the group with ischemia, 3.7% in those with normal MPS, and 3% in the group with abnormal MPS without ischemia. The independent predictors of mortality and revascularization were, respectively, a total perfusion defect greater than 6%, and an ischemic defect greater than 3%. Forty-two percent of the patients underwent MPS less than 2 years after PCI, and no significant differences were observed in relation to those who underwent it after this period.

The presence of silent ischemia in patients undergoing PCI is not uncommon, and is usually related to persistent or progressive CAD in remote vessels, rather than in the treated vessels.^8,9^ The study by de Andrade et al.^7^ demonstrated that 30% of the patients had silent ischemia, and that the 2-year period did not influence the power of MPS to predict events. However, there are no data in the literature demonstrating consistently that the diagnosis of ischemia after PCI modifies clinical outcomes.ISCHEMIA trial was designed to determine the value of the quantification of ischemia through non-invasive methods, and whether an invasive management strategy improves clinical outcomes when added to optimal medical therapy in patients with CAD and moderate or severe ischemia, but the results are not yet known.^10^ In the light of current knowledge, the presence of ischemia detected by MPS is an excellent cardiovascular risk marker, and can be a gatekeeper for invasive management strategy. In patients undergoing PCI, particularly if CAD was not fully revascularized, or if the patient did not present angina as a manifestation of CAD, MPS before the time suggested in the literature may be useful and not futile. It is up to the attending physician to consider whether the time suggested in the literature should be waited to reassess the asymptomatic patient after PCI, since the data to support this practice is not robust.
